# Simulation-Based Learning Supported by Technology to Enhance Critical Thinking in Nursing Students: Protocol for a Scoping Review

**DOI:** 10.2196/36725

**Published:** 2022-04-04

**Authors:** Hege Vistven Stenseth, Simen Alexander Steindal, Marianne Trygg Solberg, Mia Alexandra Ølnes, Andrea Mohallem, Anne Lene Sørensen, Camilla Strandell-Laine, Camilla Olaussen, Caroline Farsjø Aure, Fernando Riegel, Ingunn Pedersen, Jaroslav Zlamal, Jussara Gue Martini, Paula Bresolin, Silje Christin Wang Linnerud, Andréa Aparecida Gonçalves Nes

**Affiliations:** 1 Lovisenberg Diaconal University College Oslo Norway; 2 Faculty of Health Studies VID Specialized University Oslo Norway; 3 Faculdade Israelita de Ciências da Saúde Albert Einstein São Paulo Brazil; 4 Faculty of Health and Welfare Novia University of Applied Sciences Turku Finland; 5 Federal University of Rio Grande do Sul Porto Alegre, Rio Grande do Sul Brazil; 6 Nord University Namsos Norway; 7 Federal University of Santa Catarina Florianópolis, Santa Catarina Brazil

**Keywords:** simulation-based learning, technological supported simulation-based learning, critical thinking, nursing students, nursing education, educational approach, education, nursing

## Abstract

**Background:**

Critical thinking is a crucial skill in the nursing profession, so teaching strategies and methodology must be carefully considered when training and preparing nursing students to think critically. Studies on simulation-based learning supported by technology are increasing in nursing education, but no scoping reviews have mapped the literature on simulation-based learning supported by technology to enhance critical thinking in nursing students.

**Objective:**

The proposed scoping review aims to systematically map research on the use of simulation-based learning supported by technology to enhance critical thinking in nursing students.

**Methods:**

The proposed scoping review will use the framework established by Arksey and O’Malley and will be reported according to the PRISMA (Preferred Reporting Items for Systematic Reviews and Meta-Analyses) extension for scoping reviews. A systematic, comprehensive literature search was performed in the LILACS, ERIC, MEDLINE, EMBASE, PsycINFO, and Web of Science databases. Pairs of authors independently selected the articles by screening titles, abstracts, full-text papers, and extract data. The data will be analyzed and thematically categorized.

**Results:**

The development of a comprehensive and systematic search strategy was completed in June 2021. The database searches were performed in July 2021, and the screening of titles and abstracts was completed in September 2021. Charting the data began in February 2022. Analysis and synthesis will be performed sequentially, and the scoping review is expected to be complete by May 2023.

**Conclusions:**

The results of this proposed scoping review may identify gaps in the literature and provide an overview of research on the topic of simulation-based learning supported by technology to enhance critical thinking in nursing students. The research may identify nursing students’ reported barriers and enablers for learning critical thinking skills through simulation-based learning supported by technology, and the results may help educators enhance their educational approach through knowledge of students’ firsthand experiences and further development of successful teaching strategies in nursing education.

**International Registered Report Identifier (IRRID):**

DERR1-10.2196/36725

## Introduction

### Educational Approaches for Active Learning in Nursing Education

Active teaching methods are recognized as educational approaches by which teachers support students in the development of critical thinking (CT) [1,2]. One strategy for developing CT is to allow nursing students to actively participate in the learning process with the support of technology. Simulation-based learning (SBL) is a teaching strategy that may enhance the integration of theoretical and practical knowledge and the ability to reflect and to give and receive feedback [3,4]. Scientific evidence reveals that active learning strategies are more effective for developing CT skills for students in higher education than passive learning under traditional methods, such as lectures [2,5]. With the advent of the COVID-19 pandemic and its resultant social distancing requirements, the interest in, demand for, and use of technological solutions have increased in nursing education [6].

### Critical Thinking in Nursing Education

CT is a crucial skill and a fundamental component of nurses’ daily professional responsibilities. Nurses require CT skills to analyze, summarize, and evaluate information and initiate action. CT skills enable nurses to manage uncertainties in nursing practice and contribute to safe and effective care across diverse clinical settings [7-9]. Several definitions and terms for CT are used interchangeably in nursing studies, research, and nursing curricula [10,11]. The core components of CT are to be able to analyze, evaluate, and investigate [12]. Because there is no consensus on the definition of CT in nursing education research, research often looks to other disciplines like philosophy, psychology, and education for clear definitions [10]. A frequently cited definition in nursing studies is the one by a consensus statement of the American Philosophical Association, which defines CT as “a judgment which is purposeful and self-regulatory and results in a process of interpretation, analysis, evaluation, and inference, as well as explanation of the evidential, conceptual, methodological, criteriological, or contextual considerations upon which that judgment is based” [13]. According to Riegel and Crossetti [14], CT is driven by internal motivation, which is reflective in nature and involves self-monitoring and self-correction. This process develops a reflective judgment on what to do, believe, or make sense of in any context.

Several distinct terms are currently used in studies exploring the outcome of CT in SBL, such as *clinical problem-solving*, *clinical decision-making*, *clinical reasoning,* and *handling clinical deterioration* [4,11,15].

In this proposed scoping review, the terms *clinical decision-making*, *analytical thinking*, *creative thinking*, *problem-solving*, *reflective thinking*, *diagnostic reasoning,* and *clinical judgment* are all potential synonyms of CT. Teaching CT is the responsibility of nurse educators [16], and teaching strategies and methodology must be carefully considered to meet the purpose of preparing pre- and postgraduate nursing students to think critically and manage the uncertainty of the nursing profession [10,12,17].

### Simulation-Based Learning

Reflection and CT skills may be developed through learning activities with high-quality teaching strategies, such as SBL [7,10]. SBL facilitates learning in a safe environment with the opportunity to gain experience and practice without the risk of doing harm to the patient [3]. Bland et al [18] define SBL as “a dynamic process involving the creation of a hypothetical opportunity that incorporates an authentic representation of reality, facilitates active student engagement, and integrates the complexities of practical and theoretical learning with opportunity for repetition, feedback, evaluation, and reflection.” SBL is commonly founded on social constructivism and learning theory, which view knowledge as being constructed in a social context [19]. Within this framework, the traditional teacher-student relationship, in which knowledge is transferred from teacher to student, shifts to a learner-centered, teacher-guided approach [9,20]. SBL can potentially replicate clinical practice, in which the learner must employ clinical reasoning with cognitive, psychomotor, and affective skills [15]. According to the International Nursing Association for Clinical Simulation and Learning (INACSL) Standards Committee [21] self-monitoring, conscious reflection, and insightfulness occur in SBL through debriefing, feedback, and guided reflection. This process may help learners understand their own actual practice; identify knowledge gaps; increase competence; and support the transfer of knowledge, skills, and attitudes. Learners’ insights may be developed through conscious reflection that connects actions, thoughts, and beliefs.

In traditional SBL, high-tech modalities, including advanced simulators (eg, life-size patient manikins), replicate real patients and settings in health care [22]. In simulation research, the term *fidelity* traditionally describes the degree to which the advanced simulator looks, acts, and feels like a human being, with an emphasis on technological features and advances that enhance the physical resemblance [23].

Other simulation research focuses on different aspects of realism with a physical, semantic, and phenomenal dimension, but what constitutes realism depends on what makes sense for the individual in a given context or situation [24,25].

To enhance learning, scholars recommend focusing on learner engagement and correspondence between the simulation technology and the surroundings (the applied context) [23].

### Simulation-Based Learning Supported by Technology

Technological solutions to support SBL in nursing education are continually expanding [4], ranging from advanced physical simulators with human features and responses to computer and online games, simulation games, and virtual reality (VR). Simulation gaming for nursing education has emerged in many forms and reportedly offers potential as a teaching strategy for stimulating CT [26,27]. Producers offer specific software that enables virtual computer simulations, and there are online solutions including computer games, virtual simulations, and VR intended for nursing education. Immersive VR uses special headsets that immerse the student in a virtual world [28-30] and has the advantage of replicating the clinical environment and patient-nurse interactions in situations designed to promote specific learning outcomes [26]. Cant and Cooper [29] conclude that internet simulation measures up to other simulation approaches and will likely be a large part of the nursing curriculum in the near future.

SBL supported by technology can ensure equitable learning opportunities by providing the same content and learning environment to all students. The potential for individual training and multiple iterations through technology makes SBL resource-efficient due to its low staff costs [27,31,32]. Due to technological advances, SBL no longer requires a physical meeting space. In virtual meetings, students and teachers can discuss and reflect on dilemmas and situations experienced in simulated or clinical practice. According to the principles of metacognition, this can encourage CT. Technology-supported learning methods can stimulate dialogue between students and teachers, adjusting students’ learning focus and ensuring an accurate assessment of learning outcomes [33]. Importantly, in the context of current and future pandemics, technology provides an environment for teaching vital CT skills that is contactless and thus at low risk of spreading infectious disease [32].

### Background for the Scoping Review

A literature review by Adib-Hajbaghery and Sharifi [34] found uncertainties about the effect of SBL on the CT of nursing students and nurses. Their findings are supported by a recent systematic review that examined extant evidence of simulation’s effectiveness in promoting clinical reasoning skills in nursing education [15]. The authors of this systematic review conclude that insufficient evidence exists to form conclusions. They found a lack of substantial evidence for the cause-effect relationship of simulation training and CT due to the great heterogeneity of the studies, including diverse methods, scenarios, and measurement instruments [15,34]. The heterogeneity of studies makes it challenging to compare results and reach a consensus regarding SBL’s effect on CT. Systematic reviews have also noted a lack of comparative studies that could report a quantitative, overall effect of SBL [30,34]. A systematic review of randomized controlled trials (RCTs) found that SBL may improve the acquisition of CT knowledge as well as students’ reported satisfaction with teaching, but the authors note a lack of unambiguous evidence of SBL’s effectiveness [35].

Reviews have also examined the use of technology in nursing education and SBL with diverse outcomes. A scoping review by Duff et al [28] examined the use of online virtual simulation to enhance clinical reasoning in the education of health care professionals and found online virtual simulation to be comparable or superior to traditional simulation. However, only 3 of the 12 included studies related to nursing education.

A systematic mapping review by Plotzky et al [32] examined the use of VR in nursing education, but the review was limited to the use of VR technology from didactic and technical perspectives and did not report on the outcome of CT. According to a recent systematic review, VR provides educational outcomes similar or superior to traditional SBL practices, but the evidence is limited [36]. Another literature review concluded that most evidence indicates that virtual simulation can effectively improve skills, learning, and CT in nursing education [4], but CT was the least explored outcome, and the search used only two databases, PubMed and CINAHL. Moreover, only articles in English were included, which is an important limitation of the results.

The identified reviews did not thoroughly examine the range and use of technology in SBL to enhance nursing students’ CT skills. Furthermore, the identified reviews mainly included research presented in the English language, except two reviews that included studies in Farsi and German. A broad, comprehensive literature review, such as a scoping review that includes papers in several languages (English, Portuguese, Spanish, and the Scandinavian languages) and employs diverse research methods will enable us to examine the nature and range of the currently available research and to identify potential gaps in the research literature [37]. To our knowledge, no scoping review has examined the range of technology used in SBL and how it is used to enhance nursing students’ CT skills.

Consequently, this scoping review aims to systematically map research on the use of SBL supported by technology to enhance CT in nursing students. The results may identify potential gaps in research and inform further research on this topic.

### Identifying the Research Questions

The scoping review will answer the following research questions:

- What is the range of technology used in SBL to enhance CT skills in nursing education?
- How is technology used in SBL to enhance CT skills in nursing education?
- What do nursing students report as perceived barriers and enablers to enhance CT skills in SBL supported by technology?

## Methods

### Overview Of Method for Conducting the Scoping Review

The proposed scoping review will follow Arksey and O´Malley´s [37] framework, which includes the following steps: (1) identifying the research questions; (2) identifying relevant studies; (3) selecting studies; (4) charting the data; and (5) collating, summarizing, and reporting the results. The PRISMA (Preferred Reporting Items for Systematic Reviews and Meta-Analyses) extension for scoping reviews will guide the reporting of the proposed review [38]. The reporting of this protocol is guided by the PRISMA Protocol (PRISMA-P) [39].

### Identifying Relevant Studies

The Sample, Phenomenon of Interest, Design, Evaluation, and Research (SPIDER) framework determined the inclusion and exclusion criteria as outlined in Table 1 [40].

**Table 1:** Eligibility criteria according to the Sample, Phenomenon of Interest, Design, Evaluation, and Research (SPIDER) framework.

Criterion	Inclusion	Exclusion
Sample (S)	Papers studying undergraduate and postgraduate nursing students.	Papers studying health care students other than nursing students.
Phenomenon of interest (PI)	Using SBL^a^ supported by technology to stimulate CT^b^, clinical decision-making, analytical thinking, creative thinking, problem solving, reflective thinking, diagnostic reasoning, or clinical judgement in educational/institutional contexts. SBL supported by technology, including manikin-based, virtual reality, online virtual simulation, augmented reality, or computer-based simulation.	SBL that does not use technology. SBL using technology but not related to CT or similar concepts. SBL in clinical practice not related to education.
Design (D)	Studies with quantitative, qualitative, or mixed-methods design.	N/A^c^
Evaluation (E)	Undergraduate and postgraduate nursing students’ perspectives and experiences regarding the use of technology in SBL to stimulate CT or similar concepts.	Nurse educators’ perspectives and experiences regarding the use of technology in SBL to stimulate CT.
Research type (R)	Studies of any research type published in Portuguese, Spanish, English, Norwegian, Swedish, or Danish published in peer-reviewed journals.	Case studies, case reports, clinical guidelines, all types of reviews, and master’s and PhD theses, conference proceedings and abstracts, letters, comments, discussion editorials, and book chapters.

^a^SBL: simulation-based learning.

^b^CT: critical thinking.

^c^N/A: not applicable.

### Selecting Studies

A systematic search was conducted in the LILACS, ERIC, CINAHL, MEDLINE, EMBASE, PsycINFO, and Web of Science databases on June 28, 2021. Each database was searched from its inception. The database search will be updated approximately 3 months prior to publication.

The search strategy in Ovid MEDLINE, using Medical Subject Headings and text words, was designed by the first research librarian (author MAØ) in collaboration with the rest of the research team and embraced three elements: (1) SBL, (2) technology, and (3) nursing students and nursing education. A second research librarian (KLM) reviewed the search strategy using the Peer Review of Electronic Search Strategies (PRESS) checklist [41]. The search strategy in Ovid MEDLINE is provided in Multimedia Appendix 1. We also performed manual searches in the reference lists of the included papers. We did not perform forward tracking (citation searches). We will conduct the entire search a second time around 3 months prior to submission; most of these studies will probably be identified without using forward tracking.

MAØ exported the identified citations into EndNote to remove duplicates using the method described in Bramer et al [42], and the citations were then exported to the web application Rayyan for storage, organization, and blinding of the study selection process. A pilot test of 10% of the citations to screen titles and abstracts was performed independently by authors HVS and AAGN, who concluded that the eligibility criteria did not require modification. Pairs of authors (HVS-CFA, SCWL-SAS, MTS-JZ, AGCM-FR, PB-JGM, ALS-CSL, CO-HVS, and IP-AAGN) independently screened paper titles and abstracts to assess whether they met the inclusion criteria. When there was any doubt regarding inclusion, a third author independently assessed the full-text paper, and the decision was based on a negotiated consensus. Further, the same pairs of authors will independently assess whether the full-text papers meet the inclusion criteria. When there is any doubt regarding inclusion, a third author will independently assess the full-text paper, and the decision will be based on a negotiated consensus. The reasons for excluding full-text papers will be recorded, and the study selection process will be recorded using the PRISMA 2020 flow diagram.

### Charting the Data

A standardized data collection form will be developed in Microsoft Word for data extraction from the included papers, including authors, year, country, aim, sample, design, technology, simulation procedures, scenario design, and results related to the research question. The data collection form will be piloted by HVS and AAGN on up to five of the included papers. Their experiences will be discussed with the entire research team, and the data collection form may be revised.

Pairs of authors will extract the data, with one author extracting the data and the other checking its accuracy. Disagreement among pairs of authors will be resolved by an assessment by a third author, and agreement will be based on negotiated consensus.

### Collating, Summarizing, and Reporting the Results

HVS, SAS, MTS, and AAGN will analyze the results from the included papers and will use an inductive approach to organize the results thematically, a method previously used in scoping reviews [43,44]. The results extracted from the included papers will be read several times to identify patterns of similarities and differences related to the research questions, and these patterns will be organized in thematic groupings. The preliminary thematic groupings will be discussed with the rest of the research team, and a frequency table showing which papers appear in which thematic groupings will be created. Any new findings from the replicated search will be analyzed to see if they fit according to the thematic groups or if new thematic groupings arise.

### Ethics Approval

No ethical board approval is necessary to conduct this scoping review.

## Results

The development of a comprehensive, systematic search strategy was completed in June 2021. The database searches were performed in July 2021, and the screening of titles and abstracts was completed in September 2021. Assessment of full-text papers, charting of the data, and summarizing the results began in February 2022. We anticipate that the scoping review will be completed by May 2023.

## Discussion

The results of the proposed scoping review will identify and provide an overview of the research on using SBL supported by technology to enhance CT in nursing students. This scoping review may also identify the variety of technological solutions available for nursing education and describe how they are used to enhance the development of nursing students’ CT skills. Scoping searches have found reviews on the topic of simulation and CT in nursing education [15,30,34,35], but those reviews do not specifically report on the use of technology to support SBL to enhance CT. Reviews on SBL technologies have also been identified, which often investigate one type of technology or compare the use of technology to traditional SBL [27,29,31].

The outcome of CT is present, but not as the primary outcome for nursing students [28]. The identified reviews do not sufficiently report on the range of technology used and how technology is used in SBL to enhance CT skills in nursing students. Furthermore, the reviews do not adequately reference the outcome of enhancement of CT in nursing students. Strengths and limitations will be thoroughly examined and reported in the proposed scoping review. Limitations may be related to the inclusion criteria, by only including research studies and thus excluding grey literature. Mapping research in multiple languages may add strength to this proposed scoping review, as the exclusion of studies published in other language than English was reported as a limitation in previous scoping reviews [45].

Identifying the status of and gaps in the research in this field may contribute to future research and further the development of successful teaching strategies in nursing education. The findings may inform educators’ decisions when choosing technology to support the application of SBL, and identifying nursing students’ barriers or enablers to learning CT skills through technology-supported SBL may help educators devise their educational approaches. The results of this scoping review may also interest technology developers and guide the further development of technology-based solutions for SBL aimed at enhancing nursing students’ CT in nursing education. The results of this proposed scoping review will be disseminated through publication in relevant peer-reviewed journals in educational or nursing-specific contexts.

## Appendix

Multimedia Appendix 1 Search strategy in Ovid MEDLINE.

## Abbreviations

CT: critical thinking
INACSL: International Nursing Association for Clinical Simulation and Learning
PRESS: Peer Review of Electronic Search Strategies
PRISMA: Preferred Reporting Items for Systematic Reviews and Meta-Analyses
PRISMA-P: Preferred Reporting Items for Systematic Reviews and Meta-Analyses Protocols
SBL: simulation-based learning
SPIDER: Sample, Phenomenon of Interest, Design, Evaluation, and Research
VR: virtual reality
