# Supplementary material for: Simulation-Based Learning Supported by Technology to Enhance Critical Thinking in Nursing Students: Protocol for a Scoping Review
Source: JMIR Res Protoc. 2022 Apr 4;11(4):e36725. doi: 10.2196/36725 (PMC9016502; doi:10.2196/36725)
Supplement: Multimedia Appendix 1 [file resprot_v11i4e36725_app1.docx]

| Search date | 28.06.2021 |
| --- | --- |
| Database | Ovid MEDLINE(R) ALL 1946 to June 24, 2021 |

| 1 | simulat*.tw,kf. | 579412 |
| --- | --- | --- |
| 2 | sbl.tw,kf. | 496 |
| 3 | (manikin* or mannequin*).tw,kf. | 5107 |
| 4 | ("high-fidelity" or "low-fidelity").tw,kf. | 10525 |
| 5 | (skill* adj2 training*).tw,kf. | 10531 |
| 6 | (Standardi#ed adj3 Patient*).tw,kf. | 8754 |
| 7 | (Anatomic* adj2 Model*).tw,kf. | 2789 |
| 8 | ("Role* play*" or roleplay*).tw,kf. | 25625 |
| 9 | (skill* adj2 (Lab or labs or laborator*)).tw,kf. | 1061 |
| 10 | (Learning adj2 (Lab or labs or laborator*)).tw,kf. | 312 |
| 11 | ((Practical or practice) adj1 (Lab or labs or laborator*)).tw,kf. | 2409 |
| 12 | exp Simulation Training/ | 10182 |
| 13 | Models, Anatomic/ | 20211 |
| 14 | Role Playing/ | 2276 |
| 15 | Manikins/ | 5323 |
| 16 | or/1-15 | 652518 |
| 17 | Computer Simulation/ | 195800 |
| 18 | augmented reality/ | 391 |
| 19 | virtual reality/ | 2995 |
| 20 | vr.tw,kf. | 9096 |
| 21 | ((virtual* or augmented) adj2 (realit* or environment*1 or room* or patient* or simulation*)).tw,kf. | 21585 |
| 22 | ((web or online or computer*) adj3 simulation*).tw,kf. | 27345 |
| 23 | ("e-simulation*" or esimulation*).tw,kf. | 27 |
| 24 | 17 or 18 or 19 or 20 or 21 or 22 or 23 | 234490 |
| 25 | exp Telemedicine/ | 35203 |
| 26 | Telecommunications/ or exp Telemetry/ or Wireless Technology/ or exp Videoconferencing/ or User-Computer Interface/ or Computer assisted instruction/ or Internet-Based Intervention/ or Audiovisual Aids/ | 79477 |
| 27 | Mobile Applications/ or exp Telephone/ | 35015 |
| 28 | computers/ or microcomputers/ or computers, handheld/ or smartphone/ or minicomputers/ or exp Video Games/ | 78016 |
| 29 | (((wearable or wireless) adj2 (technolog* or electronic)) or (digital adj2 medicine) or (technolog* adj2 health) or ((mobile or internet or electronic* or robot*) adj2 (consultation* or application*1))).tw,kf. | 29172 |
| 30 | (telecommunicat* or tele-communicat* or teleconferenc* or tele-conferenc*or app or apps or app-based or mobile-based or "Short Message Service*" or sms or textmessag* or text-messag* or texting or videoconferenc* or video-conferenc* or webconferenc* or web-conferenc* or webcast* or web-cast* or webinar* or web-application* or web-based-application*).tw,kf. | 33803 |
| 31 | (phone*1 or telephon* or smartphone* or smart-phone* or cellphone* or cell-phone* or mobilephone* or mobile-phone* or "personal digital assistant*" or palmpilot* or palm-pilot* or smarthome* or smart-home* or tablet*1).tw,kf. | 159613 |
| 32 | (telemedicin* or tele-medicin* or telehealth* or tele-health* or telecare* or tele-care* or telecari* or tele-cari* or emedic* or e-medic* or ehealth* or e-health* or mhealth* or m-health* or ehomecare* or e-homecare* or e-home-care* or teleconsultation* or tele-consultation* or videoconsultation* or video-consultation* or telenurs* or tele-nurs* or teletherap* or tele-therap* or telerehab* or tele-rehab* or erehab* or e-rehab* or telemonitor* or tele-monitor*).tw,kf. | 40080 |
| 33 | high-tech*.tw,kf. | 5771 |
| 34 | (game* or gaming or gamification or gamify or videogame* or "video game*").tw,kf. | 66442 |
| 35 | ((computer or multimedia or digital* or online) adj5 (program* or education* or environment* or learn* or interface* or instruction* or based or platform* or device* or app or apps or application*)).tw,kf. | 92028 |
| 36 | technolog*.tw,kf. | 530778 |
| 37 | (touchscreen* or "touch screen*").tw,kf. | 2291 |
| 38 | ((portable or handheld or hand-held) adj1 computer*).tw,kf. | 992 |
| 39 | or/25-38 | 981637 |
| 40 | ((nurse or nursing or nurses) adj4 (student* or education or undergrad* or under-grad* or bachelor* or baccalaur* or preregistration or pre-registration or post* or practitioner* or school* or college*)).tw,kf. | 81262 |
| 41 | exp Education, Nursing/ | 85352 |
| 42 | Students, Nursing/ | 26256 |
| 43 | or/40-42 | 137858 |
| 44 | 16 and 39 | 51129 |
| 45 | 24 or 44 | 269284 |
| 46 | 43 and 45 | 1577 |
| 47 | limit 46 to (danish or english or norwegian or portuguese or spanish or swedish) | 1534 |
